# Phylosymbiosis: Relationships and Functional Effects of Microbial Communities across Host Evolutionary History

**DOI:** 10.1371/journal.pbio.2000225

**Published:** 2016-11-18

**Authors:** Andrew W. Brooks, Kevin D. Kohl, Robert M. Brucker, Edward J. van Opstal, Seth R. Bordenstein

**Affiliations:** 1 Department of Biological Sciences, Vanderbilt University, Nashville, Tennessee, United States of America; 2 Vanderbilt Genetics Institute, Vanderbilt University, Nashville, Tennessee, United States of America; 3 The Rowland Institute at Harvard, Harvard University, Cambridge, Massachusetts, United States of America; 4 Department of Pathology, Microbiology, and Immunology, Vanderbilt University, Nashville, Tennessee, United States of America; Stanford University School of Medicine, United States of America

## Abstract

Phylosymbiosis was recently proposed to describe the eco-evolutionary pattern, whereby the ecological relatedness of host-associated microbial communities parallels the phylogeny of related host species. Here, we test the prevalence of phylosymbiosis and its functional significance under highly controlled conditions by characterizing the microbiota of 24 animal species from four different groups (*Peromyscus* deer mice, *Drosophila* flies, mosquitoes, and *Nasonia* wasps), and we reevaluate the phylosymbiotic relationships of seven species of wild hominids. We demonstrate three key findings. First, intraspecific microbiota variation is consistently less than interspecific microbiota variation, and microbiota-based models predict host species origin with high accuracy across the dataset. Interestingly, the age of host clade divergence positively associates with the degree of microbial community distinguishability between species within the host clades, spanning recent host speciation events (~1 million y ago) to more distantly related host genera (~108 million y ago). Second, topological congruence analyses of each group's complete phylogeny and microbiota dendrogram reveal significant degrees of phylosymbiosis, irrespective of host clade age or taxonomy. Third, consistent with selection on host–microbiota interactions driving phylosymbiosis, there are survival and performance reductions when interspecific microbiota transplants are conducted between closely related and divergent host species pairs. Overall, these findings indicate that the composition and functional effects of an animal's microbial community can be closely allied with host evolution, even across wide-ranging timescales and diverse animal systems reared under controlled conditions.

## Author Summary

Studies on the assembly and function of host-microbiota symbioses are inherently complicated by the diverse effects of diet, age, sex, host genetics, and endosymbionts. Central to unraveling one effect from the other is an experimental framework that reduces confounders. Using common rearing conditions across four animal groups (deer mice, flies, mosquitoes, and wasps) that span recent host speciation events to more distantly related host genera, this study tests whether microbial community assembly is generally random with respect to host relatedness or "phylosymbiotic," in which the phylogeny of the host group is congruent with ecological relationships of their microbial communities. Across all four animal groups and one external dataset of great apes, we apply several statistics for analyzing congruencies and demonstrate phylosymbiosis to varying degrees in each group. Moreover, consistent with selection on host–microbiota interactions driving phylosymbiosis, transplanting interspecific microbial communities in mice significantly decreased their ability to digest food. Similarly, wasps that received transplants of microbial communities from different wasp species had lower survival than those given their own microbiota. Overall, this experimental and statistical framework shows how microbial community assembly and functionality across related species can be linked to animal evolution, health, and survival.

## Introduction

A large body of literature has documented genetic and environmental influences on the composition of host-associated microbial communities [1–10]. Although environmental factors are considered to play a much larger role than host genetics and evolutionary history [11], host influences and their functional consequences are poorly elucidated and thus require systematic study across host–microbiota systems. Several outstanding questions remain regarding the nature of host effects on microbiota assembly. Are host–microbiota associations stochastically assembled, or might there be deterministic assembly mechanisms that predict these associations? How rapidly do microbiota differences form between closely related host species, and are interspecific microbiota differences prone to decay over evolutionary time? Can host-driven assembly of the microbiota be isolated from confounding variables such as diet, age, sex, and endosymbionts? If there are microbiota differences between species, are they functional in an evolutionarily informed manner, such that mismatches between host and interspecific microbiota lead to reductions in fitness or performance, particularly when interspecific microbiota transplants are conducted between older host species pairs?

**Fig 1:**
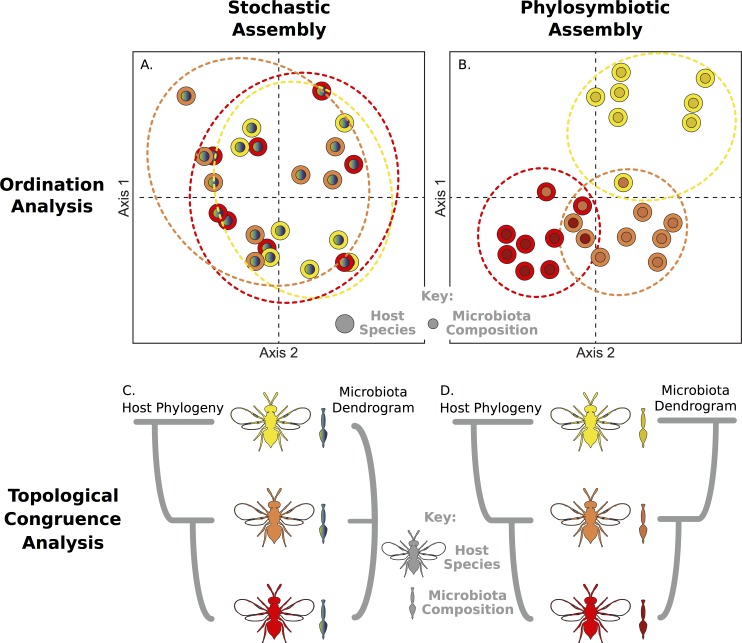
Analyses and predictions that can distinguish stochastic host–microbiota assembly from phylosymbiosis under controlled conditions. Two-dimensional ordination plots depict hypothetical microbiota similarity under (A) stochastic versus (B) phylosymbiotic models. Dashed lines represent host-specific clustering. Topological congruence analyses between host phylogeny (evolutionary relatedness) and microbial community dendrogram (ecological relatedness) depict the pattern expected for (C) stochastic versus (D) phylosymbiotic host–microbiota assembly.

If host-associated microbial communities assemble stochastically through environmental acquisition with no host-specific influence, then microbiota compositions across related host species will not differ from expectations based on random community assemblies and dispersal limitations. Therefore, in a common environment, microbiota will form independent of host species (Fig 1A), and any interspecific differences in microbiota composition would be arbitrary. In contrast, if hosts influence a sufficient amount of the composition of the microbiota, then under controlled rearing conditions, intraspecific microbial communities will structure more similarly to each other than to interspecific microbial communities (Fig 1B). Similarly, if microbial communities are randomly established or are not distinguishable with regard to host evolutionary relationships, then dendrograms illustrating beta diversity distance relationships between microbial communities will not parallel the phylogeny of the host species (Fig 1C). However, if microbial communities are distinguishable, then hosts with greater genetic divergence may exhibit more distinguishable microbiota. In this case, there will be congruence between the host phylogeny and microbiota dendrogram (Fig 1D). As this outcome is not likely due to coevolution, cospeciation, or cocladogenesis of the entire microbial community from a last common ancestor, "phylosymbiosis" was proposed as a new term that does not necessarily presume that members of the microbial community are constant, stable, or vertically transmitted from generation to generation [1,12]. Rather, phylosymbiosis refers to an eco-evolutionary pattern in which evolutionary changes in the host associate with ecological changes in the microbiota.

Phylosymbiosis leads to the explicit prediction that as host nuclear genetic differences increase over time, the differences in host-associated microbial communities will also increase. Indeed, phylosymbiosis has been observed in natural populations of sponges [13], ants [10], bats [14], and apes [15,16]. However, other studies on termites [17], flies [18–20], birds [21], and mice [22] have not observed strict patterns of phylosymbiosis or host-specific microbial signatures. In natural population studies, determining the forces driving phylosymbiosis is equivocal, as both environmental and host effects can covary and contribute to microbiota assembly. Importantly, major effects of the environment, age, or sex may overwhelm the ability to detect phylosymbiosis. Indeed, diet is a stronger determinant of whole microbial community structure than genotype in lab-bred mice [23]. Additionally, conjecture about the formation of host-specific communities should be resolved in a wider context, especially their functional significance, as microbiotas may be inconsequential to host biology or uniquely situated for certain host genotypes and fitness. Thus, the prevalence and functional significance of phylosymbiosis is uncertain and requires reductionist approaches to discriminate among the frequently confounded variables of host, environment, development, sex, and even endosymbiont status.

Here, we quantify phylosymbiosis under laboratory conditions to control for environmental and host rearing variation. Prior investigations of phylosymbiosis have not typically controlled for these confounding variables, with the exception of male *Nasonia* wasps [1,2] and *Hydra* [5,24]. Specifically, we reared 24 species in the laboratory while controlling for sex (virgin females), age, diet, and endosymbionts, thus removing major environmental variables and isolating the contribution of host species on microbiota assembly. The experimental systems, or “host clades,” span four species of *Nasonia* parasitic jewel wasps, six species of *Drosophila* fruit flies, eight species of *Anopheles*, *Aedes*, and *Culex* mosquitoes, and six species of *Peromyscus* deer mice. An externally derived dataset with seven members of the hominid lineage [16] provides another mammalian and multigenus clade for reference and facilitates examination of natural populations in which phylosymbiosis was previously documented. Together, the five host clades include 31 distinct taxa and span a range of estimated divergence times from 0.2–108 million y. Last, we test the hypothesis that phylosymbiosis represents a functional association through a series of microbial transplants with autochthonous (intraspecific) and allochthonous (interspecific) microbiota in *Nasonia* and *Peromyscus*. We expect that an experimentally mediated disruption of phylosymbiosis will have functional costs that may lower host fitness or performance in an evolutionarily informed manner. Our findings demonstrate that a consistent set of controlled experimental and bioinformatic approaches in comparative microbiota studies can isolate host-driven phylosymbiosis.

## Results

### Host Clade Differentiates Microbial Communities

**Fig 2:**
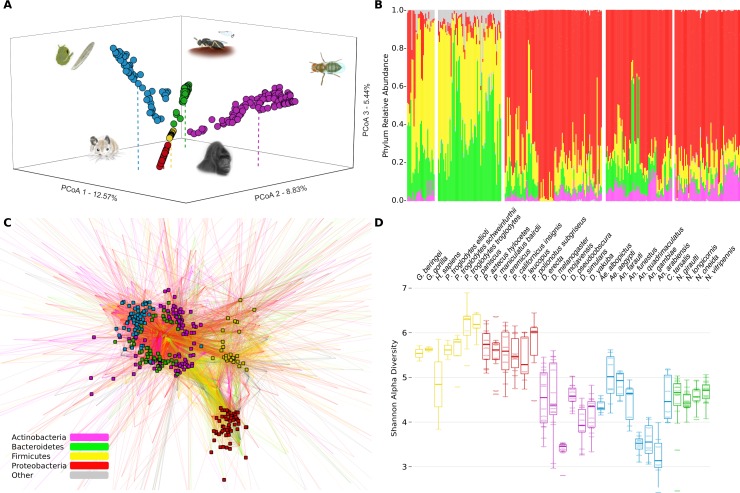
Meta-analysis of microbiota variation across five host clades. (A) PCoA analysis of Bray–Curtis ecological similarity in three dimensions based on 99% operational taxonomic unit (OTU) cutoff, with colors depicting clade of origin. (B) Phylum level relative abundance for all samples, with a key provided in C. (C) Network analysis in which small squares depict samples, with their color indicating clade of origin. Lines connect genus-level OTUs to samples and are weighted by occurrence and colored by OTU phylum. (D) Shannon alpha diversity for each host species. Small ellipses depict individual samples, and dark lines indicate the species’ median diversity. The lower and upper end of each box represent the 25th and 75th quartiles, respectively. Whiskers denote the 1.5 interquartile range. Data available at [26] in folders (A) Fig_2A, (B) Fig_2B, (C) Fig_2C, (D) Fig_2D.

Phylosymbiosis predicts that host clades will harbor distinguishable microbial communities (e.g., jewel wasps versus fruit flies versus deer mice, etc.) and that more closely related host clades will exhibit more similar microbial communities (e.g., insects versus mammals). Indeed, at a broad scale, we found that host clades harbored relatively distinct microbial communities (Fig 2A, ANOSIM, R = 0.961, *p* < 1e–6). Furthermore, there was significant microbiota differentiation between the mammalian and invertebrate host clades in the principle coordinates analysis (PCoA) (Fig 2A, ANOSIM, R = 0.905, *p* < 1e–6). The PCoA shows insect groups separating along two dimensions of a plane, with the mammals distinguished orthogonally from that plane in a third dimension, suggesting that variance in insect microbial communities is fundamentally different than that in mammals. As is well established, the gut communities of mammals were dominated by the bacterial classes Clostridia (Firmicutes) (Fig 2B, hominid 42%, *Peromyscus* 37%) and Bacteroidia (Bacteroidetes) (Fig 2B, hominid 15%, *Peromyscus* 37%), while the insect clades were dominated by Proteobacteria (Fig 2B, *Drosophila* 78%, mosquito 69%, *Nasonia* 77%). This same bacterial divide is also seen in the network analysis, with significant clustering of the insect microbial communities around Proteobacteria, and the mammal microbial communities around subsets of shared and unique Firmicutes and Bacteroidetes (G-test, *p* < 1e–6, Fig 2C). Microbial diversity as measured by the Shannon index [25] was approximately 35% higher in mammalian hosts compared to insects, indicating more diverse symbiont communities among the mammalian clades (Fig 2D; Nested analysis of variance [ANOVA]: phylum effect [mammals versus insects]: F_1,302_ = 419.82, *p* < 0.001; clade effect nested within phylum: F_3,298_ = 18.46, *p* < 0.001; species effect nested within clade and phylum: F_26,272_ = 7.94, *p* < 0.001).

We implemented a random forest classifier (RFC) supervised learning algorithm to quantify the degree to which individual microbial communities can be classified into their respective host clade. RFC models show a strong ability to classify microbial communities to their correct host clades based on OTUs (98.5% classification accuracy) (S1 Table). Additionally, models distinguish mammals and insect samples with high accuracy (95.9% classification accuracy) (S1 Table). Cross-validation prevents overfitting by ensuring that classification accuracy is assessed using only samples excluded from model training. We also used RFC models to identify the most distinguishing bacterial taxonomic level for both interclade distinction and the divide between mammals and insects. Genera provided the strongest ability to predict host clade (99.0% classification accuracy) (S1 Table); however, the major groups of insects and mammals were better distinguished by family-level community classification (98.3% classification accuracy) (S1 Table). Taken together, these results illustrate that evolutionary relationships of the host clades broadly covary with differences in microbial communities. While differentiation of the five clades could in part be attributable to varied experimental conditions for each animal group (since they were reared separately), clustering of the vertebrate microbial communities from the insect microbial communities is independent of rearing conditions and suggests a host-assisted structuring of microbial communities.

### Intraspecific Microbial Communities Are Distinguishable within Host Clades

**Fig 3:**
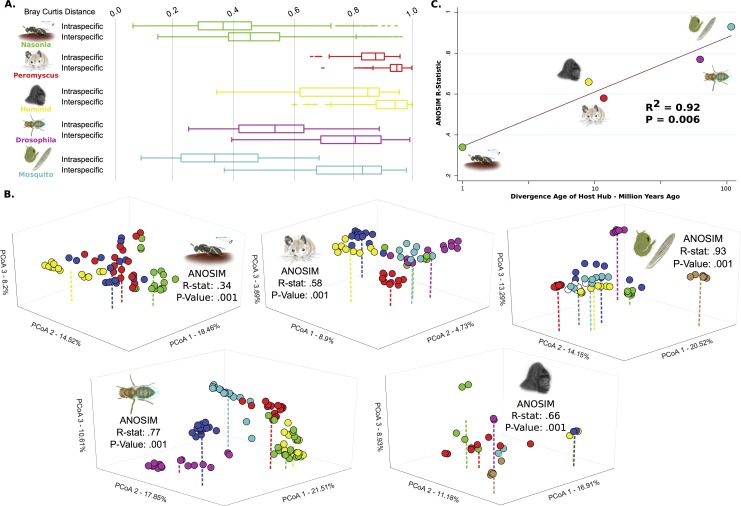
Intraspecific versus interspecific microbial community variation within and between host clades. (A) Box-and-whisker plot of intraspecific and interspecific Bray–Curtis distances between samples for each clade. Boxes represent the 25th to 75th quartiles, with the central line depicting the group median and whiskers showing the 1.5 interquartile extent. (B) PCoA of Bray–Curtis distances with first three most distinguishing dimensions shown. Colors represent different species and correspond to the colors in Fig 4. (C) Regression analysis measuring the correlation between the evolutionary age of host clade divergence on a log scale and the ANOSIM R-values of intraspecific microbiota distinguishability from part B for each host clade. Data available at [26] in folders (A) Fig_3A_&_S1, (B) Fig_3B, (C) Fig_3C.

Phylosymbiosis predicts that an individual’s microbial community will exhibit higher similarity to communities of the same host species than to those from different host species. The degree of similarity can be variable but should correlate with genetic relatedness of the host species. Pairwise comparisons of beta diversity distances between all individuals within each host clade reveal that the average distance between microbial communities within a species is always less than between species (S1 Fig). Summarized beta diversity also reveal lower intraspecific versus interspecific distances, with significant differences observed for all clades (Fig 3A, Each dataset: Mann–Whitney U, *p* < 1e–6).

We next evaluated intraspecific microbiota clustering through Bray–Curtis beta diversity interrelationships with PCoA and statistically assessed the strength of interspecific microbiota distinguishability with ANOSIM (Fig 3B). Visualization of the first three principle components revealed that individual samples clustered around their respective species’ centroid position. In all host clades, each host species harbored significantly distinguishable microbial communities (Fig 3B, ANOSIM *p* < 0.001 for all host clades). Notably, the ANOSIM R-values of interspecific microbiota distinguishability within a host clade positively correlated with the maximal age of divergence of the species in the host clades (Fig 3C, Regression Analysis Log Transformed Clade Age, R^2^ = 0.92, *p* = 0.006; Untransformed Clade Age, R^2^ = 0.70, *p* = 0.048). Thus, host clades with higher total divergence times between species had stronger degrees of microbiota distinguishability, while less diverged host clades exhibited less microbiota distinguishability. For example, with an estimated host divergence time of 108 million y [27], mosquitoes showed the greatest distinguishability of their microbiota. Conversely, in *Nasonia* jewel wasps, which only diverged between 200,000 and 1 million y ago [28], the relative strength of clustering was less distinct but still statistically significant. The three intermediate aged clades showed corresponding intermediate levels of clustering: *Drosophila* had an estimated divergence time of 62.9 million y [29], hominids diverged 9 million y ago [30], and *Peromyscus* diverged 11.7 million y ago [31]. Therefore, the phylosymbiotic prediction that host species will exhibit significant degrees of specific microbiota assembly was supported in these observations, even under highly controlled conditions in the laboratory models. Microbiota specificity was maintained among very closely related and very divergent species, and a connection was observed between the magnitude of host genetic divergence and microbiota similarity.

### Supervised Classification: Microbiota Composition Predicts Host Species

As microbiota clustering was supported within species across all five animal clades, it should be possible to model the strength of how well communities of bacteria predict their host species and how specific members of the microbiota affect these predictions. We therefore used RFC models trained on the microbiota of each host clade to evaluate classification accuracy (i.e., the percentage of assigning microbiota to their correct host species) and the expected predicted error (EPE, i.e., the ratio of model accuracy relative to random classification). RFC results indicated that the operational taxonomic units (OTUs) for *Drosophila* and *Peromyscus* and genus taxonomic levels for hominid, mosquito and *Nasonia* have the highest classification accuracies, with significant EPE observed for all clades (EPE > 2, S1 Table). At the genus level, the mosquito and *Drosophila* host clades exhibited the strongest results (mosquito, classification accuracy = 99.8%, EPE = 558.9; *Drosophila*, classification accuracy = 97.2%, EPE = 31.7). Other host clades demonstrated significant but comparatively lower strength models. The reduced predictive power of these models may be due to a number of factors, such as a lower number of host species (*Nasonia*, classification accuracy = 88.7%, EPE = 13.4), uneven sample representation from each species (hominid, classification accuracy = 53.4%, EPE = 2.1), and lower sequencing coverage (*Peromyscus*, classification accuracy = 61.4%, EPE = 2.5).

To determine the most distinguishing genera of the bacterial community, we examined the resulting loss of model classification accuracy when each genus was excluded from RFCs (S2 Table). Distinguishability within the *Drosophila*, *Nasonia*, and mosquito clades was driven primarily by genera in Proteobacteria, which represent five (14.0% model accuracy), seven (11.3% model accuracy), and eight (18.2% model accuracy) of the top ten genera, respectively. Three of the ten most distinguishing genera in *Drosophila* females are from the Acetobacteraceae family (9.5% model accuracy), previously recognized to be “core” microbiota members [19,32]. Three of the twenty most distinguishing genera in *Nasonia* females were closely related symbionts from the Enterobacteriaceae family (genera: *Proteus*, *Providencia*, *Morganella*; 3.1% model accuracy), consistently found in our previous studies of *Nasonia* males [1,2]. Eight genera from the phylum Proteobacteria dominate mosquito female distinguishability, primarily three Gammaproteobacteria of the order Pseudomonadales (8.2% model accuracy), and three Betaproteobacteria of the family Comamonadaceae (5.9% model accuracy). Hominid interspecific distinguishability was driven by the phylum Firmicutes, particularly of the order *Clostridiales* that contains three of the most distinguishing genera (1.5% model accuracy). The genus *Allobaculum* conferred nearly double the distinguishing power of any other bacteria in *Peromyscus* (3.8% model accuracy), and it is associated with low-fat diet, obesity, and insulin resistance in mice [33]. As may be expected, genera of the abundant phyla Firmicutes and Bacteroidetes dominated the majority of distinguishability in *Peromyscus* (10.6% model accuracy), but genera from Proteobacteria in the family Helicobacteraceae comprised four of the top eleven genera (4.4% model accuracy). Overall, microbiota composition can be used to predict host species with high accuracy, and genera commonly observed in other studies of these host clades underlie interspecific distinguishability.

### Phylosymbiosis Is Common within Host Clades

The major prediction of phylosymbiosis is that phylogenetic relatedness will correlate with beta diversity relationships of microbial communities among related host species. Microbiota dendrograms were constructed by collapsing individual samples to generate an aggregate microbial community for each species and then by comparing relationships of their beta diversity metrics. The matching cluster and Robinson–Foulds tree metrics were utilized to calculate host phylogenetic and microbiota dendrogram topological similarity, with normalized distances ranging from 0.0 (complete congruence) to 1.0 (complete incongruence; [34]). Matching cluster weights topological congruency of trees, similar to the widely used Robinson–Foulds metric [34,35]. However, matching cluster takes into account sections of subtree congruence and therefore is a more refined evaluation of small topological changes that affect incongruence. Significance of the matching cluster and Robinson–Foulds analyses was determined by the probability of randomized bifurcating dendrogram topologies yielding equivalent or more congruent phylosymbiotic patterns than the microbiota dendrogram. Additionally, using the same methodology, matching cluster and Robinson–Foulds metrics were evaluated for Bray–Curtis, unweighted UniFrac [36], and weighted UniFrac [36] beta diversity dendrograms at both 99% and 97% clustered OTUs (S2 Fig). The cytochrome oxidase I (COI) gene was used to construct the phylogeny for each host clade, which compared well to established phylogenetic or phylogenomic trees for all species included in the study (*Nasonia* [27]; *Drosophila* [28]; hominids [29]; mosquitoes [26]). *Peromyscus* was further resolved with an additional marker (arginine vasopressin receptor 1A [AVPR1A]) to reflect the latest phylogenetic estimates [37,38].

**Fig 4:**
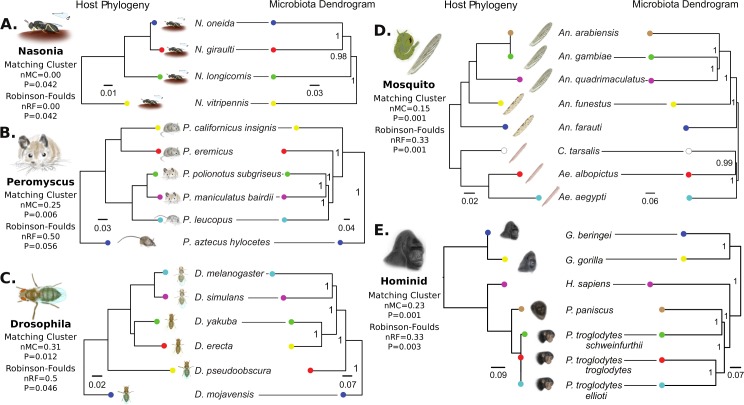
Phylosymbiosis between host phylogeny and microbiota dendrogram relationships. Topological congruencies are quantified by the normalized Robinson–Foulds (RF) metric, which takes into account symmetry in rooted tree shape on a scale from 0 (complete congruence) to 1 (incomplete incongruence). The normalized matching cluster (MC) metric is a refined version of the RF metric that sensitively accounts for incongruences between closely related branches. Horizontal lines connect species whose position is concordant between host phylogeny and microbiota dendrogram based on 99% OTU cutoffs, therefore requiring no topological shift to demonstrate phylosymbiosis. Data available at [26] in folder Fig_4.

*Nasonia* female wasps exhibited an equivalent phylogenetic tree and microbial community dendrogram, representing exact phylosymbiosis (*Nasonia* wasps, Fig 4A). These results parallel previous findings in *Nasonia* males [1,2]. Despite congruency, the *Nasonia* clade has limited topological complexity with only four species, therefore resulting in a relatively marginal significance. Mice also show nearly perfect congruence, with the exception of *Peromyscus eremicus* (Fig 4B). *Drosophila* fruit flies (Fig 4C) showed the lowest topological congruency but were still moderately significant. Four of the six species show correct topological relationships, while the microbial community relationships of *Drosophila pseudoobscura* and *D*. *erecta* are topologically swapped. These results are different from previous findings in *Drosophila* that utilized a different experimental design, set of taxa, and sequencing technology [19]. However, the evidence for phylosymbiosis is tentative in *Drosophila* as, unlike other clades, there is no significant congruence for either unweighted or weighted UniFrac metrics (S2 Fig). Previous studies detected no pattern of phylosymbiosis across *Drosophila* species [19], which could be attributed to *Drosophila’s* constant replenishment of microbes from the environment [18,20] or the dominance by the bacterial genus *Acetobacter*, which is important for proper immune and metabolic development [19]. The two additional clades, mosquitoes and hominids, showed significant phylosymbiosis (Fig 4D and 4E). Specifically, the mosquitoes showed accurate separation of *Culex* and *Aedes* genera from *Anopheles*, and the topological departures from phylosymbiosis appeared in two of the bifurcations between closely related species. The hominid microbial community dendrogram reflects the correct branching of *Gorilla* from *Homo sapiens*, followed by bonobos and chimpanzees, with the exception that one of the chimpanzee subspecies grouped more closely with the bonobo lineage. These results are similar to previous observations that the relationships of the microbial communities parallel those in the host phylogeny [16]. With the exception of *Drosophila*, which yielded variable evidence for host–microbiota congruence, significant degrees of phylosymbiosis were observed across clades with varying tree similarity metrics and microbiota beta diversity analyses.

### Phylosymbiosis Represents a Functional Association

Microbiota–host distinguishability and topological congruence does not strictly imply that the phylosymbiotic associations are fitness directed, though it naturally follows that a particular host species may be more ideally suited for an autochthonous versus allochthonous microbiota. We therefore performed a series of microbial transplants to test the prediction that inoculated microbiota from a different species would decrease aspects of host performance or fitness in contrast to inoculated microbiota from the same species. Moreover, if there is selection on host–microbiota interactions such that microbiotas are uniquely or better situated for resident host backgrounds, then transplanted microbiota from a divergent species could drive more pronounced reductions in host functions than transplanted microbiota from a closely related species.

**Fig 5:**
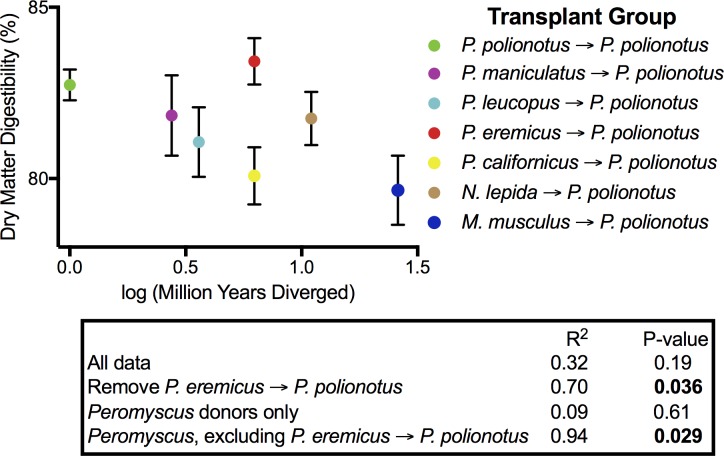
Effects of allochthonous and autochthonous microbial communities on the digestive performance of recipient mice. Dry matter digestibility is calculated as (g dry food ingested–g dry feces produced) / g dry food ingested. Divergence times between *P*. *polionotus* and donor species were determined from previously published phylogenies [37,38]. Points represent mean values ± standard error for each group (*n* = 5–6 recipients per group). Data available at [26] in folder Fig_5_&_S4.

In *Peromyscus*, we followed a previously established protocol [39] to transplant the microbial communities from six rodent donor species into a single recipient species, *P*. *polionotus*, as well as a control group in which the microbial communities from *P*. *polionotus* were introduced to intraspecific individuals of *P*. *polionotus*. Inventories of fecal microbiota from donor and recipient mice revealed that portions of the donor microbiota successfully transferred. The estimated amount of transplanted OTUs and their relative abundance ranged from 6.5%–26.2% and 11.4%–40.7%, respectively, when analyzed at the 99% OTU cutoff level. Variation in the transfer of foreign microbes was dependent on donor species and its divergence from the recipient species (S3 Fig). We then measured dry matter digestibility, or the proportion of food material that is digested by the animal. Consistent with selection on host–microbiota interactions, mice that were inoculated with microbial communities from more distantly related hosts exhibited decreased dry matter digestibility (Fig 5). These results were only significant when the group receiving feces from *P*. *eremicus* donors was removed (Fig 5). Notably, the microbiota of *P*. *eremicus* is not congruent with our predictions of phylosymbiosis (Fig 4). Thus, only the taxa showing phylosymbiosis exhibited the functional trend with digestibility. Distantly related donor species (*Neotoma lepida* and *Mus musculus*) did not drive significance, as the correlation remained statistically significant when investigating only *Peromyscus* donors (excluding *P*. *eremicus*; Fig 5).

In the most extreme cases in which mice were inoculated with the microbial communities from *P*. *californicus* or *M*. *musculus*, there was approximately a 3% decrease in dry matter digestibility, which is on par with the decrease in digestibility observed as a result of helminth infections in *Peromyscus* [40]. Animals must consume more food to meet energy demands when faced with decreases in digestibility. Indeed, mice inoculated with microbial communities from *P*. *californicus* or *M*. *musculus* exhibited significantly higher food intakes than the control group (S4 Fig; Tukey’s honest significant difference (HSD) test: *p* = 0.001 for *P*. *californicus* to *P*. *polionotus*; *p* = 0.044 for *M*. *musculus* to *P*. *polionotus*). The mice inoculated with the microbes from *P*. *eremicus* performed just as well, if not better, than the control groups in terms of dry matter digestibility (Fig 5) but still had slightly higher food intakes (S4 Fig).

**Fig 6:**
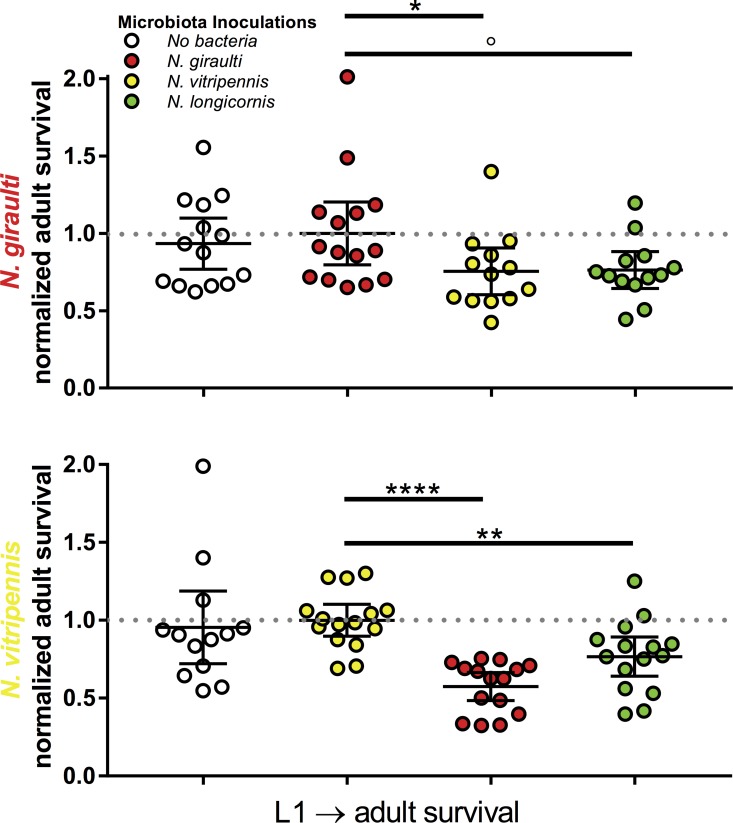
Effects of allochthonous and autochthonous microbial communities on the survival of *Nasonia* wasps. (A) Normalized larval-to-adult survival of *N*. *giraulti* wasps harboring no, self, or foreign microbiota. (B) Normalized larval-to-adult survival of *N*. *vitripennis* wasps harboring no, self, or foreign microbiota. Adult survival is calculated as number of adults in a transwell / number of first instar larvae in a transwell. Adult survival was normalized to the average survival of the autochthonous microbiota transplantation. Circles represent individual transwell samples, and the dashed line represents the average survival of the autochthonous microbiota transplantation normalized to 1; error bars represent 95% confidence intervals. Mann–Whitney U statistics, °*p* < 0.1, *p < 0.05, ** *p* < 0.01, and **** *p* < 0.0001. Data available at [26] in folder Fig_6.

In *Nasonia*, we used an *in vitro* rearing system to transplant heat-killed microbial communities from three *Nasonia* donor species into larvae of *N. vitripennis* or *N*. *giraulti* [41]. We then measured the survival of the recipients from first instar larva to adulthood. In both *N*. *vitripennis* and *N*. *giraulti* hosts, interspecific microbiota transplantations exhibited significant decreases in survival to adulthood when compared to intraspecific microbial transplantations (Fig 6). Specifically, *N*. *giraulti* with a *N*. *vitripennis* microbiota yielded a 24.5% average survival decrease in comparison to a *N*. *giraulti* microbiota (Fig 6A, Mann–Whitney U, *p* = 0.037). Interestingly, *N*. *giraulti* with a microbiota from the more closely related *N*. *longicornis* exhibited a similar but nonsignificant survival reduction (23.7%, Fig 6A, Mann–Whitney U, *p* = 0.086). *N*. *vitripennis* with a *N*. *giraulti* or *N*. *longicornis* microbiota exhibited a 42.6% (Fig 6B, Mann–Whitney U, *p* < 0.0001) and 23.3% (Fig 6B, Mann–Whitney U, *p* = 0.003) average survival decrease in comparison to a *N*. *vitripennis* microbiota, respectively (Fig 6A, Mann–Whitney U, *p* < 0.0001). Comparisons were also made between noninoculated hosts and those inoculated with interspecific backgrounds (*N*. *giraulti* background: *N*. *vitripennis* inoculum *p* = 0.07, *N*. *longicornis* inoculum *p* = 0.26; *N*. *vitripennis* background: *N*. *giraulti* inoculum *p* = 0.001, *N*. *longicornis* inoculum *p* = 0.15).

## Discussion

Under phylosymbiosis, host-associated microbial communities form, in part, as a result of interactions with the host rather than through purely stochastic processes associated with the environment. Specifically, we predicted that given closely related animals reared in controlled environments, the relationships of the microbiota would be congruent with the evolutionary relationships of the host species. Previous evidence for phylosymbiosis under controlled regimes existed in *Nasonia* [1,2] and *Hydra* [24], and wild populations of sponges [13], ants [10], and apes [15,16] also exhibited this pattern. Here, in a comprehensive analysis of phylosymbiosis in a diverse range of model systems, we report the widespread occurrence of this pattern under strictly controlled conditions as well as a functional basis in the context of host digestive performance in mice and survival in wasps. These results represent the first evidence for phylosymbiosis in *Peromyscus* deer mice, *Drosophila* flies, a variety of mosquito species spanning three genera, and *Nasonia* wasp females with the inclusion of *N*. *oneida*. Previous studies in *Nasonia* measured male phylosymbiosis and did not include *N*. *oneida* [1,2]. By rearing closely related species from the same host clade in a common environment, and by controlling age, developmental stage, endosymbiont status, and sex, the experiments rule out confounding variables that can influence microbiota relationships in comparative analyses. Eliminating these variables is important because they often substantially correlate with interspecific differences. Thus, our findings demonstrate that a uniform experimental and bioinformatic methodology can excavate host effects on phylosymbiosis from other potentially confounding variables in comparative microbiota studies.

We observed marked differences in microbial diversity and community structure between mammalian and invertebrate host clades. Mammalian communities were more diverse and dominated by Bacteroidetes and Firmicutes, while insect-associated communities were less diverse and primarily dominated by Proteobacteria. These results are consistent with previous microbial inventories conducted in mammals and insects [6,42]. Together, these findings suggest large-scale differences in the host–microbiota interactions between mammals and insects. These differences across host phyla could be due to a variety of possibilities, including host genetics, diet, age, and rearing environment.

To remove confounding variables that structure host–microbiota assemblages and to rigorously test phylosymbiosis, we utilized an experimental design within four host clades that isolated the effects of host evolutionary relationships from other effects (i.e., diet, age, rearing environment, sex, endosymbionts). We found that host species consistently harbored distinguishable microbiota within each host clade. Additionally, we found significant degrees of congruence between the evolutionary relationships of host species and ecological similarities in their microbial communities, which is consistent with the main hypothesis of phylosymbiosis. These results importantly expand previous evidence for this eco-evolutionary pattern and demonstrate that related hosts reared under identical conditions harbor distinguishable microbial assemblages that can be likened to microbial community markers of host evolutionary relationships. It is conceivable that recently diverged species (i.e., those younger than several hundred thousand years) would have less genetic variation and fewer differences in microbiota composition. Furthermore, divergent hosts may have vast differences in physiology that overwhelm the likelihood of observing phylosymbiosis. Surprisingly, we observed phylosymbiosis to varying degrees in all host clades, and the age of clade divergence positively correlates with the level of intraspecific microbiota distinguishability. Thus, as host species diverge over time, microbial communities become more distinct [1,12], and the limits of detecting phylosymbiosis may occur at extreme scales of incipient or ancient host divergence times.

The mechanisms by which phylosymbiosis is established requires systematic investigation. Perhaps the most apparent regulator of host–microbiota interactions is the host immune system. A previous study of phylosymbiosis in *Hydra* demonstrated that antimicrobial peptides of the innate immune system are strong dictators of community composition, and expression of antimicrobial peptides are necessary for the formation of host-specific microbiota [5]. Furthermore, genome-wide association studies in humans [43], mice [8], and *Drosophila* [44] have identified a large immune effect in which host immune genes can explain variation in microbial community structure. Interestingly, host immune genes often exhibit rapid evolution and positive selection compared to genes with other functions [45,46]. While this trend is often explained by the host–pathogen arms race [45], it is also likely due to host evolutionary responses for recruiting and tending a much larger collection of nonpathogenic microbes.

Other host pathways may also underlie the observed species-specific microbiota signatures. Hosts produce glycans and mucins on the gut lining that may serve as biomolecular regulators of microbial communities [47,48]. For example, knocking out the gene for α1–2 fucosyltransferase inhibits production of fucosylated host glycans on the gut surface and significantly alters microbial community structure [49]. Additional knockout studies have demonstrated the roles of circadian clock genes [50], microRNAs [51], and digestive enzymes [52] in determining microbial community structure. These various physiological systems might also interact with one another and may have even evolved in tandem to regulate microbial community structure.

Alternatively, rather than hosts “controlling” their microbiota, microbes may be active in selecting which host niches to colonize. For example, hosts have been compared to ecological islands, where environmental selection of the microbiota through niche availability may occur [53]. However, given the large number of studies that demonstrate the role of microbes in improving host performance [54], we find it unlikely that hosts would assume a solely passive role in these interactions. An elegant study allowed microbial communities from various environments (soil, termite gut, human gut, mouse gut, etc.) to compete within the mouse gut [55]. This study found that a foreign community of the human gut microbiota exhibited an early competitive advantage and colonized the mouse gut first. Later, the mouse gut microbiota dominated and outcompeted the human gut microbiota [55]. Thus, community assembly is not a monolithic process of host control but likely a pluralistic combination of host control, microbial control, and microbe–microbe competition. In this context, both population genetic heritability and community heritability measurements of the microbiota will be useful in prescribing the varied genetic influences of a foundational host species on microbiota assembly [56].

The acquisition route of microbes could also influence our understanding of phylosymbiosis. If phylosymbiosis is observed when the microbiota is acquired horizontally from other hosts, the environment, or some combination of the two, then phylosymbiosis is presumably influenced by host-encoded traits such as control of or susceptibility to microbes. However, maternal transmission of microbes is argued to be a common trend in animals [57]. For example, sponges exhibit vertical transmission of a diverse set of microbes in embryos [58]. Transmission of full microbial communities is unlikely in most systems, given that the communities of developing animals tend to exhibit markedly lower diversity and distinct community structure compared to adults [1,59,60]. Thus, it is improbable that phylosymbiotic relationships are explained simply by community drift over host evolutionary divergence. There could be a subset of microbial taxa that are more likely to be transmitted from mother to offspring that in turn affect what other microbes colonize. For instance, in humans, the family Christensenellaceae is situated as a hub in a co-occurrence network containing several other gut microbes and has a significant population genetic heritability [61]. When *Christensenella minuta* was introduced into the guts of humanized mice, the microbial community structure was significantly altered [61]. This microbe, as well as others, can therefore be likened to a keystone taxa or "microbial hub" that can impact community structure despite low abundance [61–63]. Thus, one could hypothesize that phylosymbiotic relationships in some systems may be driven by host transmission of microbial hubs that determine whole community structure through ensuing microbe–microbe interactions. However, further work is needed to test this hypothesis.

The congruent relationships between hosts and associated microbial communities are likely maintained through their positive effects on host performance and fitness but could be neutral or harmful as well. While the importance and specificity of hosts and microbes in bipartite associations has been demonstrated on host performance [64], it is unclear whether such effects commonly occur for hosts and their complex microbial communities. If they exist, disruption of phylosymbiosis via hybridization or microbiota transplants should lead to reduced fitness or performance. For instance, hybridization experiments demonstrate negative interactions or "hybrid breakdown" between host genetics and the gut microbiota that drives intestinal pathology in house mice [65] and severe larval lethality between *N. vitripennis* and *N*. *giraulti* wasps [2]. Furthermore, transplant experiments show that all microbes are not equal for the host. An early study demonstrated that germ-free rabbits inoculated with a mouse gut microbiota exhibited impaired gastrointestinal function compared to those given a normal rabbit microbiota [66]. Together, these functional studies and others suggest that interactions between hosts and their microbiota are not random and instead occur at various functional levels.

Here, we add an evolutionary component to these ideas by demonstrating that microbial communities from more evolutionarily distant hosts can be prone to more pronounced reductions in host performance or fitness. Specifically, *Peromyscus* deer mice inoculated with microbial communities from more distantly related species tended to exhibit lower food digestibility. The exception to this trend was the *P*. *eremicus* to *P*. *polionotus* group, which did not exhibit any decrease in digestibility. It should be noted that *P*. *eremicus* also did not follow phylosymbiosis (Fig 4B), which may explain the departure from our expected trend in digestibility. For example, deviations from phylosymbiosis could be due to a microbial community assembly that is inconsequential to host digestibility. Therefore, transferring a nonphylosymbiotic community between host species may not yield performance costs.

An alternative explanation for our results could be that hosts are acclimated to their established microbiota, and the introduction of foreign microbiota either elicits a host immune response or disrupts the established microbiota, thus decreasing digestibility. One technique to distinguish between adaptation and acclimation would be to conduct experiments in germ-free *P*. *polionotus* recipients. However, the derivation of germ-free mammals is a difficult and expensive process [67] and has not been conducted for *Peromyscus*. Earlier studies utilizing germ-free mammals demonstrate that microbial communities from evolutionarily distant hosts negatively impact gastrointestinal function [66] and immune development [68], thus supporting our hypothesis of functional matching between host and the gut microbiota.

Additionally, among very closely related species, *Nasonia* exposed to interspecific microbiota have lower fitness than those exposed to intraspecific microbiota. While this experiment utilized heat-killed bacteria to avoid shifts in the microbiota composition during media growth, the protocol is sufficient to test the predictions of phylosymbiosis. First, isolated microbial products can exert drastic effects on eukaryotic partners. For example, a sulfonolipid purified from bacteria can induce multicellularity in choanoflagellates [69]. Additionally, the insect immune system can respond with strain-level specificity to heat-killed bacteria [70]. Therefore, we hypothesize that each *Nasonia* host species evolved to the products of their own gut microbiota rather than those of gut microbiota from related host species. Together, results from the *Peromyscus* and *Nasonia* functional experiments reveal the importance of host evolutionary relationships when considering interactions between hosts and their gut microbial communities and ultimately the symbiotic processes that can drive adaptation and speciation [71,72]. The molecular mechanisms underlying the functional bases of phylosymbiosis in various systems demand further studies

Overall, we have established phylosymbiosis as a common, though not universal, phenomenon under controlled rearing with functional effects on host performance and survival. It is worth emphasizing again that this term is explicit and different from many other similar terms, such as coevolution, cospeciation, cocladogenesis, or codiversification [73]. While cospeciation of hosts and specific environmentally or socially acquired microbes—e.g., hominids and gut bacterial species [74] or the bobtail squid and *Vibrio* luminescent bacteria [75]—could contribute in part to phylosymbiosis, concordant community structuring with the host phylogeny is not dependent on parallel gene phylogenies but instead on total microbiota compositional divergence. Phylosymbiosis does not assume congruent splitting from an ancestral species because it does not presume that microbial communities are stable or even vertically transmitted from generation to generation [1,12]. Rather, phylosymbiosis predicts that the congruent relationships of host evolution and microbial community similarities could have varied assembly mechanisms in space and time and be newly assembled each generation (though see our discussion of transmission routes above). Moreover, the findings here imply that across wide-ranging evolutionary timescales and animal systems, there is a functional eco-evolutionary basis for phylosymbiosis, at least under controlled conditions.

It may be difficult to detect phylosymbiosis in natural populations because of extensive environmental variation that overwhelms the signal. We suggest that one way to potentially overcome this challenge is to start with laboratory-controlled studies that identify (i) phylosymbiotic communities and (ii) the discriminating microbial taxa between host species. Resultantly, investigations can test whether these microbial signatures exist in natural populations, albeit perhaps in a smaller fraction of the total microbiota that is mainly derived by environmental effects. Another advantage of controlled studies is that the functional effects, both positive and negative, of a phylosymbiotic community assembly can be carefully measured in the context of host evolutionary history.

## Materials and Methods

### Ethics Statement

Procedures involving functional microbiota transplants in *Peromyscus* mice were approved by the University of Utah Institutional Animal Care and Use Committee under protocol 12–12010. Mice obtained from the *Peromyscus* Genetic Stock Center were reared under IACUC approved protocols, and only fecal samples were directly utilized. While our paper contains data for several primate species, this data was conducted by another research group, has been previously published, and is now publicly available. Thus, there was no requirement of approved protocols for the primate species.

### *Nasonia* Husbandry and Sample Collection

*Nasonia* were reared as previously described [2]. Four strains were used: *Nasonia vitripennis* (strain 13.2), *N*. *longicornis* (IV7U-1b), *N*. *giraulti* (RV2x(u)), *N*. *oneida* (NAS_NONY(u)). To collect individuals for microbiota analysis, virgin females were sorted as pupae into sterile glass vials and collected within the first 24 h of eclosing as adults. Subsequently, they were rinsed with 70% ETOH for 2 min, a 1:10 bleach solution for 2 min, followed by two rinses in sterile water. Individuals were then placed in 1.5 ml tubes and flash frozen in liquid nitrogen. They were then stored at –80°C until DNA extractions. Fifty individuals were collected per strain.

### *Drosophila* Husbandry and Sample Collection

Nine strains of *Drosophila* were obtained from the University of California San Diego *Drosophila* Species Stock Center. Six strains were used in the microbiome analysis because they were *Wolbachia*-free: *Drosophila melanogaster* (Strain Dmel, stock number 14021–0248.25), *D*. *simulans* (Dsim, 14021–0251.195), *D*. *yakuba* (Dyak, 14021–0261.01), *D*. *erecta* (Dere, 14021–0224.01), *D*. *pseudoobscura* (Dpse, 14011–121.94), and *D*. *mojavensis* (Dmow, 15081–1352.22). The three strains that tested positive for *Wolbachia* (method described below) were: *D*. *sechellia* (14021–0248.25), *D*. *ananassae* (14021–0371.13), and *D*. *willistoni* (14030–0811.24). All strains were reared on a cornmeal media (*Drosophila* Species Stock Center: http://stockcenter.ucsd.edu/info/food_cornmeal.php) with a sterile Braided Dental Roll (No. 2, Crosstex, Atlanta, Georgia, US) inserted into the surface of the media. All stocks were incubated at 25°C with a 12-h light–dark cycle and monitored every 24 h. Every 14 d, stock vials were cleared of any emerged adults, and 6 h later, ten virgin females and three males were transferred to new food vials. This conditioning on the same food was done for five generations before setting up media vials for sample collection. For each of the six strains, five virgin females were mated with two males and allowed to oviposit for 24 h; afterwards, the parents were removed and the vials were incubated as per above.

After 12 d, vials were cleared and virgin females were collected every 4–6 h over a 36-h period. All females were rinsed with 70% ETOH for 2 min, a 1:10 bleach solution for 2 min, followed by two rinses in sterile water. Individual adult flies were then placed in 1.5 ml tubes and flash frozen in liquid nitrogen. They were then stored at –80°C until DNA extractions. Approximately 25–30 virgin adult females were collected per strain.

### Mosquito Husbandry and Sample Collection

Mosquitoes were acquired from the Malaria Research and Reference Reagent Resource Center as eggs on damp filter paper within 24 h of being laid. Eight strains were used: *Anopheles funestus* (strain name FUMOZ), *An*. *farauti s*.*s*. (FAR1), *An*. *quadrimaculatus* (GORO), *An*. *arabiensis* (SENN), *An*. *gambiae* (MALI NIH), *Aedes aegypti* (COSTA RICA), *Ae*. *albopictus* (ALBO), and *Culex tarsalis* (YOLO F13). Eggs were floated in 350 ml of sterile water with 1.5 ml of 2% yeast slurry and autoclaved within a sterile and lidded clear plastic container. Containers were enclosed within a larger sterile clear container and placed inside an incubator set at 25°C with a 12-h light–dark cycle and monitored every 24 h. After 48 h, the hatched larvae were sorted out and 100–150 of each species were placed in new sterile water (150 ml) with 30 mg of powdered koi food (Laguna Goldfish & Koi all season pellets). Water level was maintained at 150 ml, and larvae were fed 30 mg of powdered koi food every day for a total of 13 d. All pupae were discarded (frozen and autoclaved) on day 10, and new pupae were collected every 12 h on day 11, 12, and 13. Water samples were also collected and frozen for microbial analysis on day 11.

To collect individuals for microbiota analysis, pupae were sorted according to sex, and all females were rinsed with 70% ETOH for two min, then 1:10 bleach solution for two min, followed by two rinses in sterile water. Individual pupae were then placed in 1.5 ml tubes and flash frozen in liquid nitrogen. They were then stored along with their corresponding water sample at –80°C until DNA extractions. Ten to 25 individuals were collected per strain.

### *Peromyscus* Husbandry and Sample Collection

Fecal samples were collected from the *Peromyscus* Genetic Stock Center at the University of South Carolina. Six stock species of *Peromyscus* were used: *P*. *maniculatus* (stock BW), *P*. *polionotus subgriseus* (PO), *P*. *leucopus* (LL), *P*. *californicus insignis* (IS), *P*. *aztecus hylocetes* (AM), and *P*. *eremicus* (EP). All mice were reared using their standard care practices at the stock center on the same mouse chow diet. Cages were cleaned at regular intervals for all species, and all species were caged within the same facility. Individuals from nonmating cages of females (five to six per cage) were used for collections.

Fecal pellets were collected on a single morning from individual mice directly into a sterile tube and placed on dry ice before being stored at –80°C for 24 h. Samples were then shipped overnight on dry ice and again stored at –80°C until DNA extractions. One to three pellets from 15 individuals were collected per strain.

In order to eliminate the introduction of confounding factors and exclude any subjects that had a pinworm infection at the time of sample collection, we conducted a screen to confirm the pinworm status of each mouse. Pinworm status was confirmed by PCR. Primers utilized to amplify the 28S rDNA D1 and D2 domains of multiple pinworm species were developed and confirmed with positive DNA samples of *Syphacia obvelata* and *Aspiculuris tetraptera* (received from the Feldman Center for Comparative Medicine at the University of Virginia). The C1 primer 5ʹ-ACCCGCTGAATTTAAGCAT-3ʹ and the D1 primer 5ʹ-TCCGTGTTTCAAGACGG-3ʹ were amplified under the following reaction conditions: 94°C for 1 min; 35 cycles of 94°C for 30 s, 55°C for 30 s, 72°C for 30 s; and a final elongation time at 72°C for 2 min. The resultant samples were then visualized on a 1% agarose gel. Of the 84 fecal specimens analyzed, 8 of the samples showed amplification at 750 bp corresponding to the expected amplification size of the pinworm DNA sequence. For confirmation, the 750 bp bands were extracted using a Wizard Gel Extraction Kit (Promega Corporation, Madison, Wisconsin, US) and sequenced (GENEWIZ, Inc, New Jersey, US). Sequence results confirmed the presence of *Aspiculuris tetraptera* infection, and these 8 samples and were excluded from further analysis.

### *Wolbachia* Screens of Stock Insect Lines

The presence or absence of *Wolbachia* was checked using two replicates of three individuals per species. DNA extraction was performed with PureGene DNA Extraction Kit (Qiagen), and fragments of the 16S rDNA gene were PCR amplified using primer set WolbF and WolbR3 [76]. Only stock strains that were *Wolbachia* negative were used in the experiments.

### Insect DNA Extraction

Individual insects (and the mosquitoes’ corresponding water samples) were mechanically homogenized with sterile pestles while frozen within their collection tube. The samples were then thawed to room temperature for 30 s and flash frozen again in liquid nitrogen with additional mechanical homogenization. The samples were finally processed using the ZR-Duet DNA/RNA MiniPrep Kit (Zymo Research, Irvine, California, US). Samples were then quantified using the dsDNA BR Assay kit on the Qubit 2.0 Fluorometer (Life Technologies).

### DNA Isolation from Mouse Samples

The PowerSoil DNA isolation kit (Mo Bio Laboratories, Carlsbad, California, US), was utilized to extract DNA from 20 mg of mouse fecal material per sample according to manufacturer’s protocol after being mechanically homogenized with sterile pestles while frozen within their collection tube. Samples where then quantified using the dsDNA BR Assay kit on the Qubit 2.0 Fluorometer.

### PCR, Library Prep, and Sequencing

Total genomic DNA was quantified using dsDNA HS Assay kit on the Qubit. Using two μl of DNA, a 20 μl PCR reaction of 28S general eukaryotic amplification was conducted on each sample, with only 25 cycles. Products were purified using Agencourt AMPure XP, quantified using the dsDNA HS Assay kit on the Qubit, and compared to the amount of 16S amplification from the same DNA volume and PCR reaction volume as previously described [2]. PCR amplification of the bacteria 16S rRNA was performed with the 27F 5ʹ-AGAGTTTGATCCTGGCTCAG-3ʹ and 338R 5ʹ-GCTGCCTCCCGTAGGAGT-3ʹ “universal” bacterial primers with the NEBNext High-Fidelity 2X PCR Master Mix; duplicate reactions were generated per sample, which were pooled together postamplification. For sequencing runs 1 (*Peromyscus*) and 2 (*Nasonia*, mosquito, and *Drosophila*), 16S PCR products that were made into libraries had their concentrations normalized relative to about 1,000 ng/ml and 2,000 ng/ml of the 28S quantity for library prep respectively.

Using the Encore 384 Multiplex System (NuGEN, San Carlos, California, US), each samples’ 16S product was ligated with Illumina NGS adaptors and a unique barcode index (after the reverse adaptor). The samples were then purified using Agencourt AMPure XP and quantified using the dsDNA HS Assay kit on the Qubit. Samples were subsequently pooled.

Each pooled library was run on the Illumina MiSeq using either the MiSeq Reagent Kit V2 or V3 for paired-end reads. Run 1 was conducted at the University of Georgia Genomics Facility and run 2 was conducted at Vanderbilt Technologies for Advanced Genomics (VANTAGE).

### Sequence Quality Control

Sequence quality control and OTU analyses were carried out using QIIME version 1.8.0 [77]. Forward and reverse paired-end sequences were joined and filtered if they met the following criteria: they fell below an average Phred quality score of 25, contained homopolymer runs or ambiguous bases in excess of 6 nucleotides, or were shorter than 200 base pairs. Sequences were also removed if there were errors in the primer sequence or if barcodes contained errors and could not be assigned to a sample properly. A total of 5,065,121 reads passed quality control for the meta-analysis, with an average read length of 310 ± 48 nucleotides. *Drosophila*: 648,676 reads, average length 315 ± 23. hominid: 1,292,542 reads, average length 247 ± 38. mosquito: 664,350 reads, average length 328 ± 19. *Nasonia*: 864,969 reads, average length 322 ± 15. *Peromyscus*: 295,752 reads, average length 347 ± 12.

### OTU Analysis

Chimeric sequences were evaluated and removed using the UCHIME algorithm [78] for the intersection of de novo and GreenGenes 13_5 non-chimeras [79]. The sequences were then clustered into OTUs at 94%, 97%, and 99% similarity using the USEARCH open-reference method [80]. OTUs were mapped at the respective percent against the GreenGenes 13_5 database and screened for a minimum group size of two counts, with dereplication based on full sequences [79]. Representative sequences were chosen as the most abundant representative in each OTU cluster and aligned using GramAlign [81]. A phylogenetic tree of the representative sequences was built in QIIME [77] with the FastTree method and midpoint rooting [82]. Taxonomy was then assigned to the OTU representatives with the UCLUST method against the GreenGenes 13_5 database [79]. OTU tables were constructed in QIIME [77] and sorted by sample IDs alphabetically.

### Sample and OTU Quality Control

OTU tables were screened to remove any OTUs classified as chloroplast, unassigned, and *Wolbachia*. Individual samples were assessed for low sequence coverage affecting community profiles and diversity as well as for processing errors based on minimum count thresholds assessed against group means. Following rarefaction, counts were subsequently chosen as the highest rarefaction number allowed by the smallest sample’s count representation in each respective clade and the meta-analysis. Alpha diversity was measured using Shannon and Chao1 metrics generated with the QIIME alpha_rarefaction script. Plots of alpha diversity at a range of rarefied levels were used to assess and remove samples with low diversity.

### Meta-Analysis

The PCoA (Fig 2A) components for the meta-analysis were constructed using the QIIME jackknifed_beta_diversity script. The OTU table first underwent rarefaction, followed by the computation of Bray–Curtis beta diversity distances for each rarefied table. PCoA plots of the first three coordinate dimensions were generated using a custom Python script. Individual samples are each depicted as a point and are colored by host clade of origin.

The community profile (Fig 2B) for the meta-analysis was generated using a custom Python script and BIOM tools [83]. OTU tables were first converted to relative abundance for each sample, and bacterial taxonomy was collapsed at the class level. Bacterial classes were sorted alphabetically, and a stacked bar chart representing the relative abundance for each sample was constructed.

The network analysis (Fig 2C) was visualized using Cytoscape [84]. OTU tables were first collapsed by bacterial taxonomy at the genus level, and QIIME’s make_otu_network script was used to construct connections between each bacterial genus to individual hosts based on relative abundance. Network files were then imported into Cytoscape, where the network was computed using an edge-weighted force directed layout. Nodes were colored by host clade, and connections were colored by key bacterial phylum observed in high abundance (i.e., Actinobacteria, Bacteroidetes, Firmicutes, Proteobacteria) and gray for additional phylum.

Alpha diversity plots (Fig 2D) were prepared using the Phyloseq package [85]. OTU tables collapsed by host species were imported into Phyloseq, and the plot_richness function was used to generate box-and-whisker plots of Shannon alpha-diversity. Plots were colored by host clade of origin.

### Microbiota Dendrograms

Microbiota dendrograms were constructed using the QIIME jackknifed_beta_diversity script. OTU table counts were first collapsed by host species of origin to get representative species microbiota profiles. The pipeline script performed 1,000 rarefactions on each table and calculated Bray-Curtis beta diversity distances for each. Bray–Curtis distance matrices were UPGMA clustered to give dendrograms of interspecific relatedness. The role of 97% versus 99% OTU clustering cutoffs and weighted and unweighted UniFrac beta diversity measures (S2 Fig) were evaluated for Robinson–Foulds and matching cluster congruence with host phylogeny.

### Host Phylogenies

Host phylogenetic trees were constructed using sequences for each host species’ cytochrome oxidase gene downloaded from the NCBI. COI was chosen as a highly conserved molecular marker, and it is widely used for interspecific phylogenetic comparison [86]. Sequences were initially aligned using Muscle v3.8.31 [87]. Gap positions generated through inserts and deletions were removed, and overhanging sequence on 5ʹ and 3ʹ ends were trimmed. Models of molecular evolution were evaluated using jModelTest v2.1.7 [88], and the optimal model was used for final alignment and tree building in RaxML v8.0.0 [89]. The *Nasonia* and *Peromyscus* clades were carried out using the same methodology—except for final alignment and tree building in PhyML v3.0 [90]—and for *Peromyscus* the AVPR1A gene was concatenated with COI to further resolve the phylogeny. All trees are concordant with well-established phylogenies from literature references noted in the Results section.

### Robinson–Foulds and Matching Cluster Congruency Analyses

Quantifying congruence between host phylogeny and microbiota dendrogram relationships (Fig 4) was carried out with a custom Python script and the TreeCmp program [91]. The topologies of both trees were constructed, and the normalized Robinson–Foulds score [35] and normalized matching cluster score [34] were calculated as the number of differences between the two topologies divided by the total possible congruency score for the two trees. Next, 100,000 random trees were constructed with the same number of leaf nodes, and each was compared to the host phylogeny. The number of trees which had an equivalent or better score than the actual microbiota dendrogram were used to calculate the significance of observing that topology under stochastic assembly. Normalized results of both statistics have been provided to facilitate comparison. Matching cluster and Robinson–Foulds *p*-values were determined by the probability of 100,000 randomized bifurcating dendrogram topologies yielding equivalent or more congruent phylosymbiotic patterns than the microbiota dendrogram.

### Intraspecific Versus Interspecific Beta Diversity Distances

Within each clade, the Bray–Curtis distances calculated by the jackknife_beta_diversity script (Fig 3A) were separated by those that compared microbiota within a host species and those that compared between host species. The box-and-whisker plots were constructed in Python. Coloring indicates host clade of origin, and all intraspecific and interspecific distances are represented for each clade. These distances were then compared between the groups using a nonparametric, two-tailed Mann–Whitney U test implemented in SciPy [92,93].

### ANOSIM Clustering

To evaluate intraspecific clustering (Fig 3B), the ANOSIM test was used to calculate the distinguishability of Bray–Curtis distances based on species of origin. Bray–Curtis distance matrices were generated using the QIIME jackknifed_beta_diversity script on tables of individuals rarefied 1,000 times. The QIIME script compare_categories was used to calculate ANOSIM scores using the Bray–Curtis distance matrix and host species as categories. 1,000 permutations were used to calculate the significance of clustering for each clade. Three-dimensional PCoA plots were generated in Python using components generated from Bray–Curtis distance matrices in QIIME, and the first three components are shown. Points are colored by host species within each clade, and colors correlate with the species labels in Fig 4 for reference.

### Correlation of ANOSIM Clustering and Clade Age

A general linear regression was performed to test the correlation between age of clade origin and the intraspecific clustering measured through ANOSIM R-statistic scores. Cladogenesis Age was Log10 transformed to normalize the distance scale between samples (1, 10, 100 MYA). The regression was carried out in Stata v12.0 to determine the coefficient (R^2^) and significance (*p*-value).

### Random Forest Analyses

OTU tables were first collapsed at each bacterial taxonomic level (i.e., phylum… genus) using the QIIME script summarize_taxa. Then, both the raw OTU table and each collapsed table underwent ten rarefactions to an even depth using the QIIME script multiple_rarefactions_even_depth. RFC models were constructed with the supervised_learning script for 1,000 rounds of ten-fold Monte Carlo cross validation on each table. At each level, the results were collated and averages were taken for the ten rarefied tables. Host species were used as the category for RFC model distinguishability, testing the ability to assign samples to their respective host species. The average class error for each clade was subtracted from 100 to get the percent accuracy of the models at each taxonomic level. The same methodology was used for constructing RFC models for the meta-analysis, with the only exception being that host species, host clade, and vertebrate or invertebrate categories were tested for distinguishability.

### Microbiota Transplants

#### Peromyscus

We tested the effects of allochthonous microbial communities on host performance by conducting a series of microbial transplants from various donor rodent species into a single recipient species, the oldfield mouse (*Peromyscus polionotus*). We obtained virgin, female *Peromyscus* species (*P*. *polionotus*, *P*. *maniculatus*, *P*. *leucopus*, *P*. *eremicus*, *P*. *californicus*) from the *Peromyscus* stock center. We also obtained three female individuals of *Neotoma lepida* (*Neotoma* is the sister genus of *Peromyscus*) from Dr. M. Denise Dearing (University of Utah). Additionally, we obtained six female individuals of outbred *Mus musculus* from Dr. Wayne Potts (University of Utah). The founding animals of this colony were collected from near Gainesville, Florida, US, and the animals have been randomly bred in captivity for roughly 13 generations and are still highly outbred [94,95]. All rodent species were maintained on powdered laboratory rodent chow (Formula 8904, Harlan Teklad, Madison, Wisconsin, US) except for woodrats, which were fed powdered rabbit chow (Formula 2031, Harlan Teklad, Madison, Wisconsin, US), given that woodrats are herbivorous. All procedures involving rodents were approved under the University of Utah Institutional Animal Care and Use Committee protocol #12–12010.

To conduct microbial transplants, we followed a protocol that was previously established to transplant the microbiota from *Neotoma lepida* into *Rattus norvegicus* [39]. First, donor feces were collected from three to six individuals of each donor species by placing rodents in wire-bottom metabolic cages overnight and collecting feces the next morning. Feces were then ground with a mortar and pestle and mixed into powdered laboratory chow (Formula 8904, Harlan Teklad, Madison, Wisconsin, US) at a ratio of 15% w/w. Recipient animals (five to six individuals per group) were fed food containing feces of a particular donor species for two nights. Then, recipient animals were fed normal laboratory diets for 6 d, which is a sufficient time for the clearance of transient, ingested microbes [96]. We then measured food intake and dry matter digestibility by placing animals into wire-bottom metabolic cages. Animals were presented with a known amount of powdered rodent chow overnight. The next morning, remaining food was weighed, and feces were collected, dried overnight, and weighed. Food intake was calculated as g dry food presented–g dry food remaining. Dry matter digestibility was calculated as (g dry food ingested–g dry feces produced) / g dry food ingested.

We investigated whether microbial communities from more distantly related hosts affected performance metrics in recipients. We compared food intake using ANOVA and Tukey’s HSD test across recipient groups. We also conducted correlations of dry matter digestibility and estimated divergence times based off of previously published phylogenies [37,38]. We performed correlations using both untransformed divergence times and log-transformed divergence times.

#### Nasonia

We tested the effects of allochthonous microbial communities on host survival by exposing two recipient species (*N*. *vitripennis* or *N*. *giraulti*) to a suspension of heat-killed microbes isolated from three donor *Nasonia* species (*N*. *vitripennis*, *N*. *giraulti*, and *N*. *longicornis*). We reared *Nasonia* in an in vitro rearing system [41] and inoculated germ-free larvae in 6 mm diameter transwell inserts with autochthonous microbiota, allochthonous microbiota, and sterile phosphate-buffered saline (PBS) for the first 8 d after embryo hatching. Microbiota were purified from fourth instar larvae of *Nasonia* by filtration through a 5 um filter and centrifugation at 10,000 rpm for 3 min. The pellet was suspended in a sterile PBS solution at a concentration of 5 x 10^6^ CFU of microbiota bacteria (determined by tryptic soy agar plating) per milliliter. 20 uL of this microbiota suspension was added to the transwell inserts for each of the 8 inoculation days. *Nasonia* rearing media was replaced daily just before the inoculations.

Measurements of *Nasonia* survival from first instar larvae to adulthood were determined using transwell insert images taken with an AmScope MT1000 camera. For each transwell, live larval counts were recorded 3 d post–embryo hatching. Adult counts were determined by recording the number of remaining larvae and pupae in each transwell sample 20 d after embryo hatching (5–7 d after first adult eclosion) and subtracting that number from the larval counts previously recorded. Normalized adult survival per transwell sample was calculated as the percent survival of *Nasonia* from 3 d to 20 d after embryo hatching divided by the average percent survival of the autochthonous microbiota treatment group. We compared survival between the autochthonous and allochthonous treatment groups using Mann–Whitney U tests.

## Supporting Information

S1 Fig Comparisons of intraspecific and interspecific Bray-Curtis distances for pairwise combinations of all species. Bray-Curtis beta diversity distances were computed for all pairs of individuals within each clade from 99 percent OTUs. Colored circles denote the named species, and colors within box-and-whisker plots denote to which species it is being compared. Boxes represent the 25^th^ to 75^th^ quartiles with the central line depicting the group median, and whiskers showing the 1.5 interquartile extent. Data available at [96] in folder Fig_3A_&_S1.
(PDF)

S2 Fig Phylosymbiosis analysis for alternative beta-diversity metrics and OTU clustering cutoffs. The normalized Robinson-Foulds metric and the normalized Matching Cluster metric were used to evaluate the congruence between host phylogenies and microbiota dendrograms for Bray Curtis, Unweighted UniFrac, and Weighted UniFrac beta-diversity metrics at both 97 and 99 percent clustered OTUs. Data available at [96] in folder Fig_S2.
(PDF)

S3 Fig Fine-resolution overlap between donor and recipient microbial communities. White bars represent shared OTUs between donor and recipients and thus the possible range of transfer. Colored bars represent the portion of shared OTUs that are donor-specific and thus transfer of unique OTUs between donor and recipients. Panels (A) and (B) depict the mean ± s.e.m. percentage of OTUs. Panels (C) and (D) show the mean ± s.e.m abundance of total sequences. These analyses were conducted with OTU-picking at both 97% and 99% sequence identities. Data available at [96] in folder Fig_S3.
(TIFF)

S4 Fig Effects of allochthonous versus autochthonous microbial communities on the food intake of recipient mice. Divergence times between *P*. *polionotus* and donor species were determined from previously published phylogenies [38, 39]. Points represent mean values ± s.e.m. for each group (n = 5–6 recipients per group). Data available at [96] in folder Fig_5_&_S4.
(TIFF)

S1 Table Table of Random Forest accuracy in classifying the microbiota by host species in each host clade, and by host species, clade, and mammal or invertebrate taxonomy in the meta-analysis. Models were generated using OTUs or abundance collapsed by bacterial taxonomy. Red boxes highlight the highest classification accuracy. Ten-fold cross validation assessed the percent classification accuracy for test sets excluded from model training.
(XLSX)

S2 Table Table of Random Forest model mean decrease in accuracy when genera are excluded from classification of the microbiota in each host clade. Random Forest models were generated using genera collapsed bacterial taxonomies. Genera are ordered by those that contribute the most accuracy to the model to those that contribute the least accuracy to the model, measured in the form of decrease in model accuracy when a genus is excluded from model construction. Standard deviations of mean decrease in model accuracy are also provided.
(XLSX)

S3 Table Tables of microbiota taxon in the meta-analysis with varying abundance between host clades or between vertebrates and invertebrates. The meta-analysis OTU table was collapsed at each bacterial taxonomic level (Phylum to Genus), and converted to relative abundance. Kruskal-Wallis tests were performed on microbial taxon within each table, testing for differences in the mean abundance across host clades or vertebrates and invertebrates. The results were sorted from high to low significance of p-values, which are provided alongside False Discovery Rate and Bonferroni corrected p-values. Mean abundances of each taxon within host clades or vertebrates and invertebrates are provided as a heatmap, with dark blue indicating high abundance, light blue centered at the 5% most abundant values and fading to white for low abundance or non-existent taxon.
(XLSX)

## Abbreviations

ANOVA: analysis of variance
AVPR1A: arginine vasopressin receptor 1A
COI: cytochrome oxidase
EPE: expected predicted error
HSD: honest significant difference
OTU: operational taxonomic unit
PCoA: principal coordinates analysis
RFC: random forest classifier
