# Correction: Phylosymbiosis: Relationships and Functional Effects of Microbial Communities across Host Evolutionary History

**DOI:** 10.1371/journal.pbio.1002587

**Published:** 2017-01-09

**Authors:** Andrew W. Brooks, Kevin D. Kohl, Robert M. Brucker, Edward J. van Opstal, Seth R. Bordenstein

Fig 4 is incorrect. The authors have provided a corrected version which corrects the connecting lines in panels D and E. The figure legend and conclusion remain the same.

**Fig 4:**
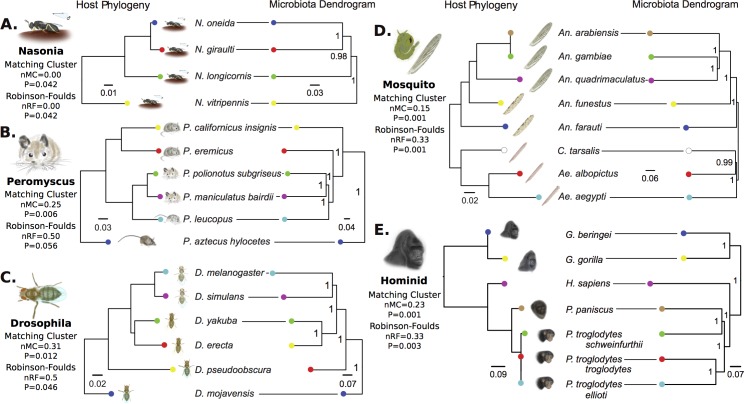
Phylosymbiosis between host phylogeny and microbiota dendrogram relationships. Topological congruencies are quantified by the normalized Robinson–Foulds (RF) metric, which takes into account symmetry in rooted tree shape on a scale from 0 (complete congruence) to 1 (incomplete incongruence). The normalized matching cluster (MC) metric is a refined version of the RF metric that sensitively accounts for incongruences between closely related branches. Horizontal lines connect species whose position is concordant between host phylogeny and microbiota dendrogram based on 99% OTU cutoffs, therefore requiring no topological shift to demonstrate phylosymbiosis. Data available at [26] in folder Fig_4.
