# Supplementary figures and images for: Phylosymbiosis: Relationships and Functional Effects of Microbial Communities across Host Evolutionary History
Source: PLoS Biol. 2016 Nov 18;14(11):e2000225. doi: 10.1371/journal.pbio.2000225 (PMC5115861; doi:10.1371/journal.pbio.2000225)

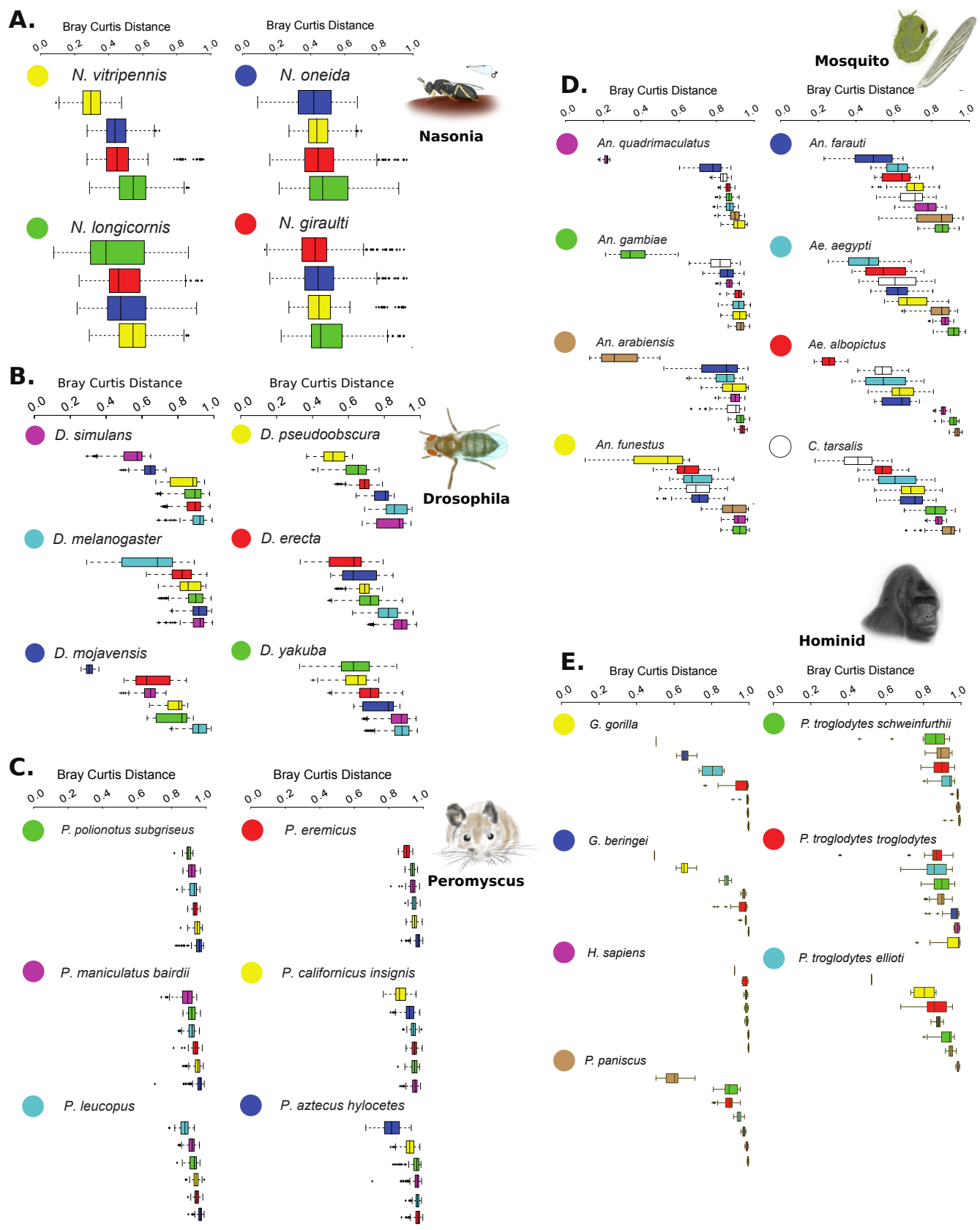

Supplement: S1 Fig — Bray-Curtis beta diversity distances were computed for all pairs of individuals within each clade from 99 percent OTUs. Colored circles denote the named species, and colors within box-and-whisker plots denote to which species it is being compared. Boxes represent the 25th to 75th quartiles with the central line depicting the group median, and whiskers showing the 1.5 interquartile extent. Data available at [96] in folder Fig_3A_&_S1. (PDF) [file pbio.2000225.s001.pdf]

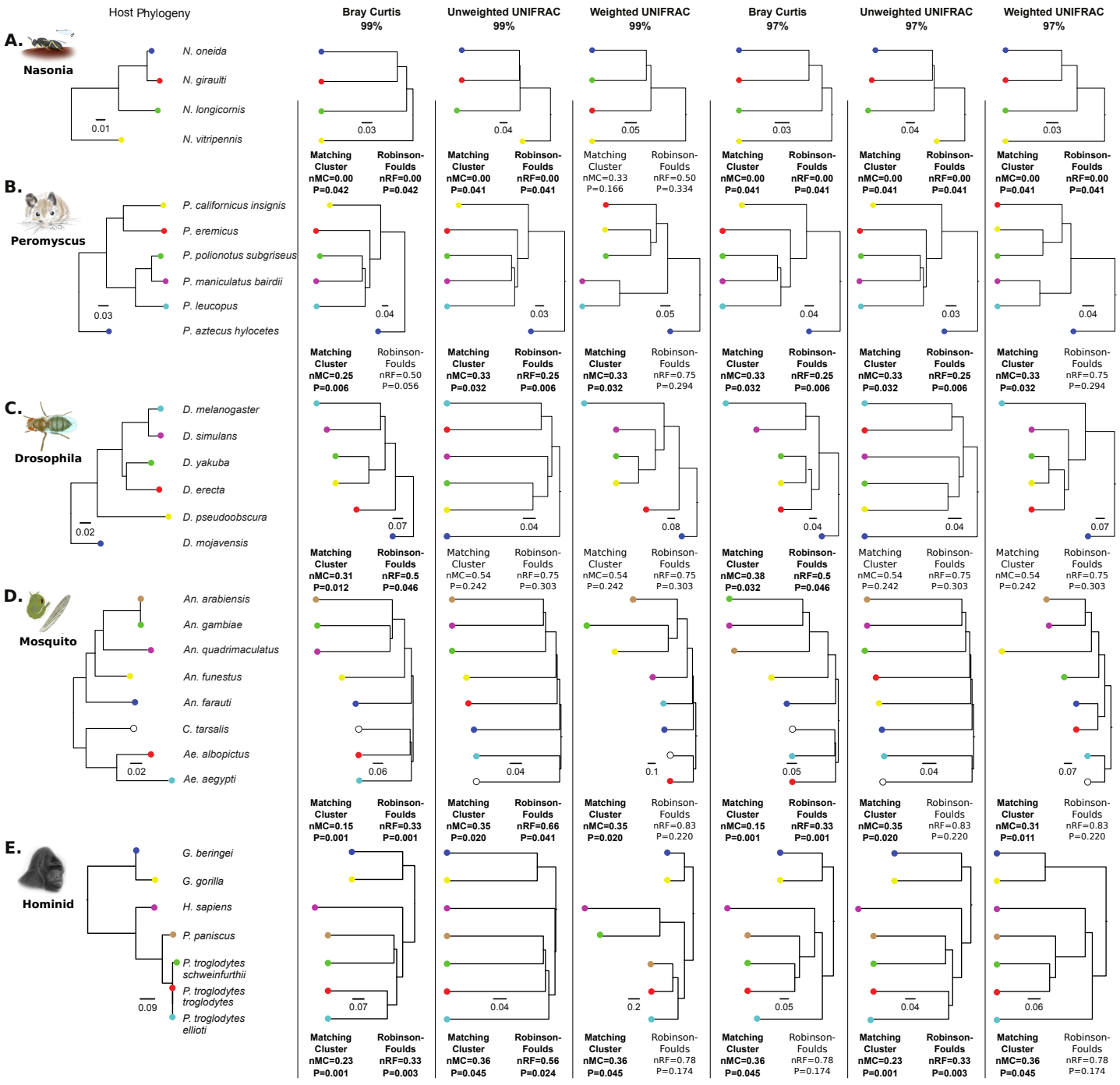

Supplement: S2 Fig — The normalized Robinson-Foulds metric and the normalized Matching Cluster metric were used to evaluate the congruence between host phylogenies and microbiota dendrograms for Bray Curtis, Unweighted UniFrac, and Weighted UniFrac beta-diversity metrics at both 97 and 99 percent clustered OTUs. Data available at [96] in folder Fig_S2. (PDF) [file pbio.2000225.s002.pdf]

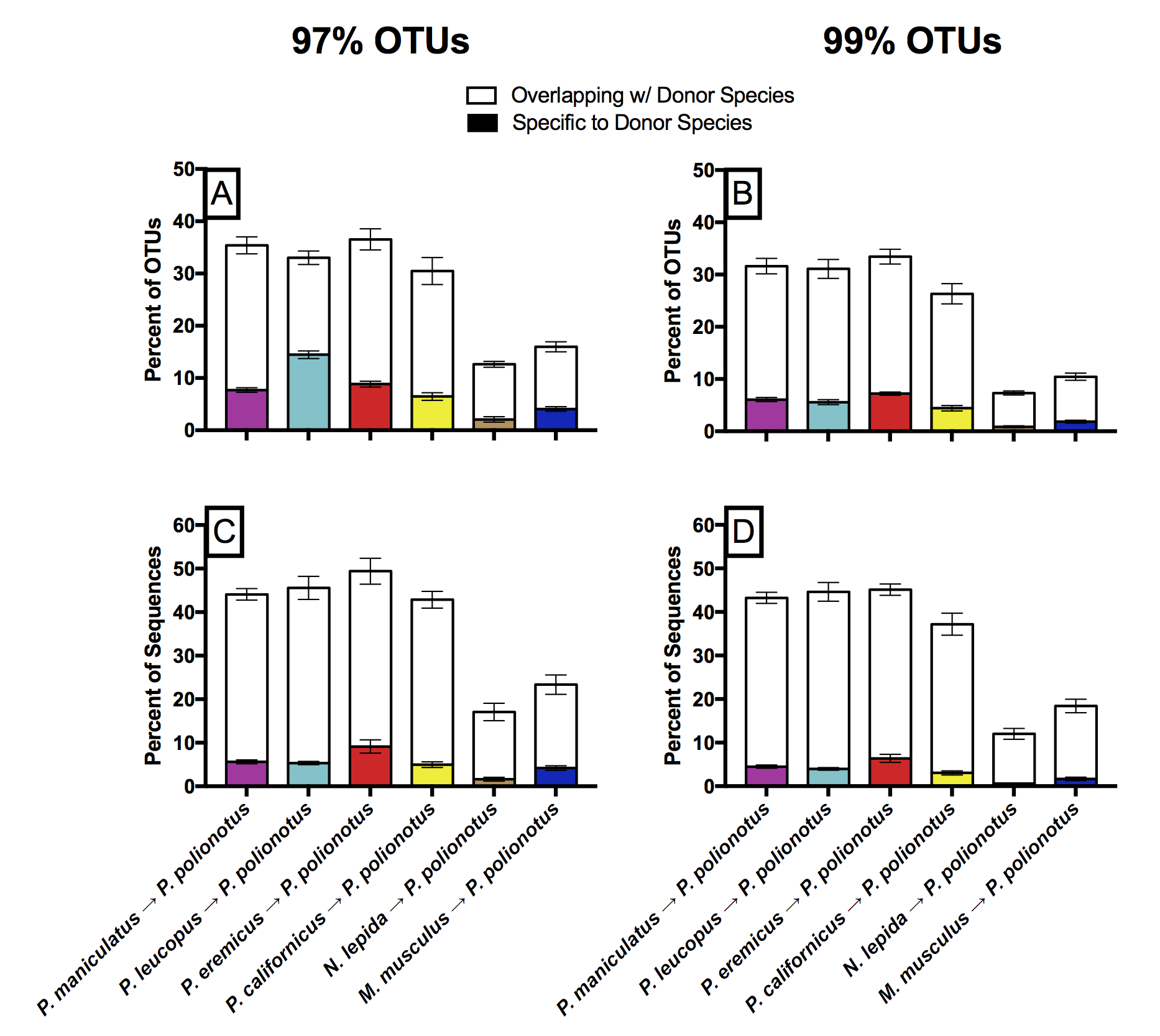

Supplement: S3 Fig — White bars represent shared OTUs between donor and recipients and thus the possible range of transfer. Colored bars represent the portion of shared OTUs that are donor-specific and thus transfer of unique OTUs between donor and recipients. Panels (A) and (B) depict the mean ± s.e.m. percentage of OTUs. Panels (C) and (D) show the mean ± s.e.m abundance of total sequences. These analyses were conducted with OTU-picking at both 97% and 99% sequence identities. Data available at [96] in folder Fig_S3. (TIFF) [file pbio.2000225.s003.tiff]

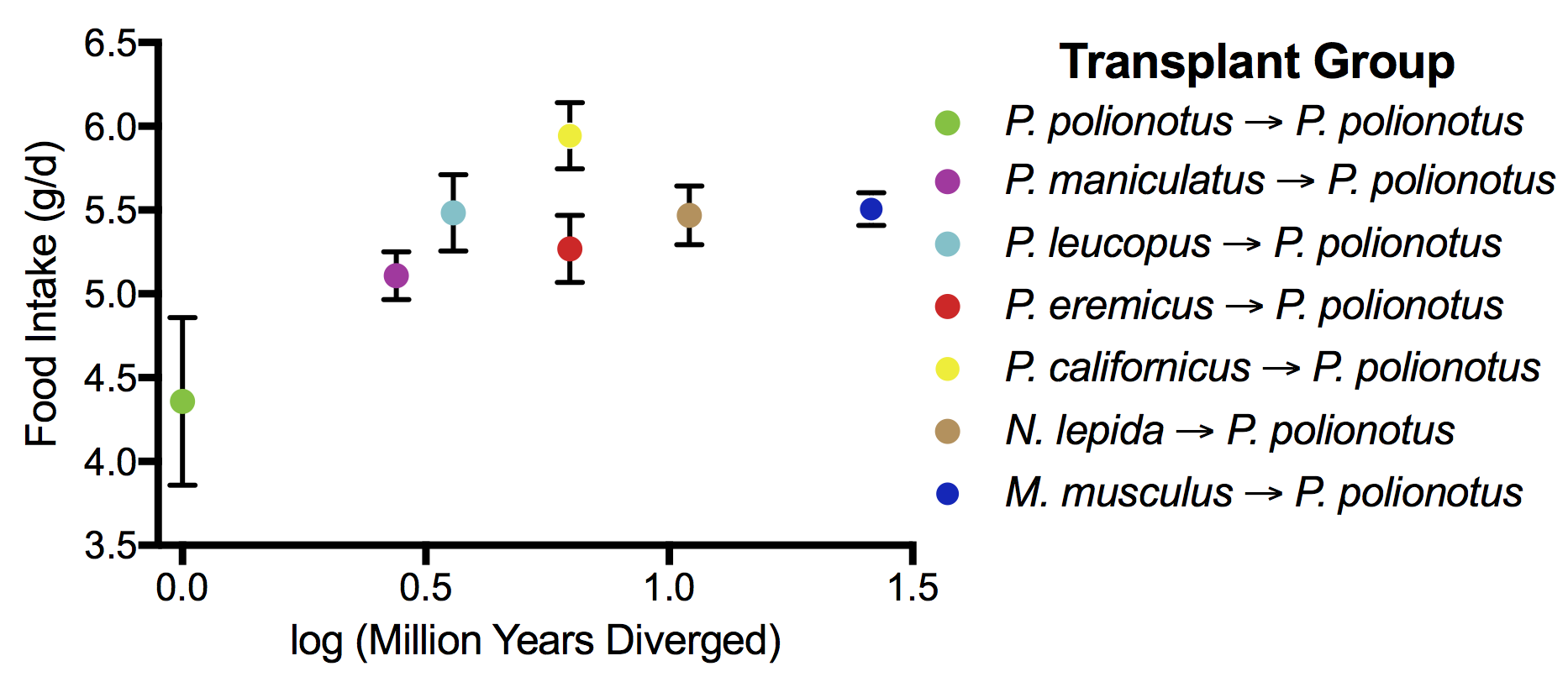

Supplement: S4 Fig — Divergence times between P. polionotus and donor species were determined from previously published phylogenies [38, 39]. Points represent mean values ± s.e.m. for each group (n = 5–6 recipients per group). Data available at [96] in folder Fig_5_&_S4. (TIFF) [file pbio.2000225.s004.tiff]
